# Radiological Evaluation of a Malignant Gastrointestinal Stromal Tumor in a Female Patient With the Coincidental Detection of Primary Breast Cancer: A Case Report

**DOI:** 10.7759/cureus.33530

**Published:** 2023-01-09

**Authors:** Prasanthi R Ghanta, Rajasbala P Dhande, Gaurav V Mishra, Harshith K Gowda

**Affiliations:** 1 Department of Radiodiagnosis, Jawaharlal Nehru Medical College, Datta Meghe Institute of Higher Education and Research Centre, Wardha, IND

**Keywords:** case report, cd117, axillary lymph node metastasis, breast lump, computed tomography, ultrasonography, gastrointestinal stromal tumor (gist)

## Abstract

Gastrointestinal stromal tumors (GIST) are a rare and unique group of mesenchymal tumors arising from the gastrointestinal tract, omentum, mesentery, and retroperitoneum. Though they have certain typical radiological features that can differentiate them from epithelial tumors, it is often difficult to differentiate them from other non-epithelial tumors. Their features also vary depending on their size, site of origin, etc. When differentiation from other mesenchymal tumors on histopathology is difficult, receptor tyrosine kinase (C-KIT proto-oncogene/CD117) and gastrointestinal stromal tumor (GIST-1) discovered on GIST1 (DOG-1) expression are confirmatory. The concurrent presence of other primary cancers with GISTs has been described in the literature, among which most have been of gastrointestinal origin. Few cases of primary breast cancer in GIST have been described. Lymph nodal metastasis is rarely encountered in GIST, and metastasis to the breast is even rarer. We present a case of a 39-year-old female with non-specific symptoms who was referred for ultrasonography (USG) and computed tomography (CT) that showed a small intestinal GIST along with a breast lump and axillary lymphadenopathy that were labeled as metastases from the GIST on frozen sections; however, they were later diagnosed as primary breast cancer with axillary metastases on the histopathology and immunohistochemistry of the excision biopsy specimens post-surgery. The patient underwent surgical resection and chemotherapy.

## Introduction

Gastrointestinal stromal tract tumors (GIST) are a rare group of tumors of the gastrointestinal tract (GIT), constituting 3% of GIT tumors [1]. Receptor tyrosine kinase (C-KIT proto-oncogene/CD117) activating mutations are involved in the causation of GISTs [2]. GISTs have characteristic imaging features, making radiological investigations part of the primary diagnostic repertoire. A fluorodeoxyglucose positron emission tomography (FDG-PET) scan is useful in staging GIST to detect metastasis and in the follow-up of patients after their treatment to detect any recurrence.

They usually occur in people older than 50, with a slight male predilection. Most of the GISTs occur sporadically. However, rarely, they are associated with the Carney triad, Carney-Stratakis syndrome, or neurofibromatosis type I. Small GISTs of the esophagus are better diagnosed on endoscopy. Barium studies were initially used to look for intestinal GISTs and demonstrate their extra mucosal origin. The advent of contrast-enhanced computed tomography (CT) and magnetic resonance imaging (MRI) scans has superseded the use of barium studies in the diagnosis of GIST [3].

## Case presentation

A 39-year-old female presented with non-specific complaints of weight loss, malaise, loss of appetite, and decreased food intake for one and a half months. She complained of a small lump in the left hypochondriac region and mild pain with pressure. She also complained of a breast lump that was insidious in onset and had been progressively growing in size for one month. There was no history of vomiting, fever, melena, or jaundice. She does not have any significant past histories, family histories, or comorbidities. Laboratory investigations revealed mild anemia, slightly raised total leukocyte counts, and no rise in carcinoembryonic antigen (CEA) and cancer antigen 19-9 (CA 19-9). On physical examination, a palpable lump with mild tenderness was present in the left hypochondriac region with no signs of redness or warmth over the skin. A palpable right breast lump and hard, enlarged right axillary lymph nodes were also present.

**Figure 1:**
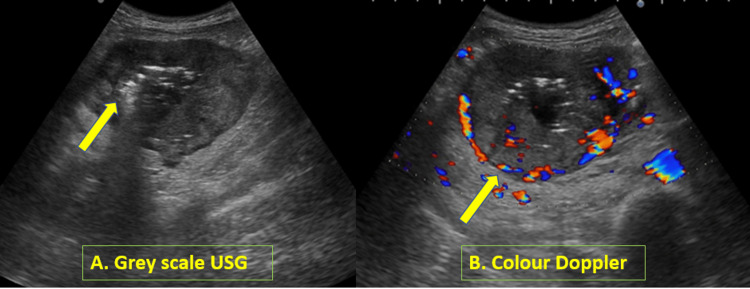
(A) B-mode grayscale ultrasound (USG) image depicting a hypoechoic lesion with central hyperechoic foci (shown by the arrow) representing air bubbles. (B) A color Doppler image showing vascularity in the lesion, more toward the periphery (shown by the arrow), with central necrosis and air foci.

The patient was referred to the radiology department for ultrasonography (USG) of the abdomen. On USG, there was a heterogeneous hypoechoic mass lesion abutting the small bowel loop with multiple central hyperechoic foci representing gas bubbles (Figure 1A). There was significantly increased vascularity in the lesion on color doppler (Figure 1B). Multiple enlarged pre- and para-aortic lymph nodes were also noted. A preliminary probable diagnosis of gastrointestinal stromal tumor (GIST) was given, and the patient was advised to undergo further evaluation with a triple-phase contrast-enhanced computed tomography (CT) scan. The USG of the breast mass showed a well-defined hypoechoic lesion with smooth margins that resembled an intraparenchymal lymph node or benign lesion. However, the enlarged axillary lymph nodes showed suspicious features like irregular margins, loss of fatty hilum, and increased vascularity on USG and Doppler.

**Figure 2:**
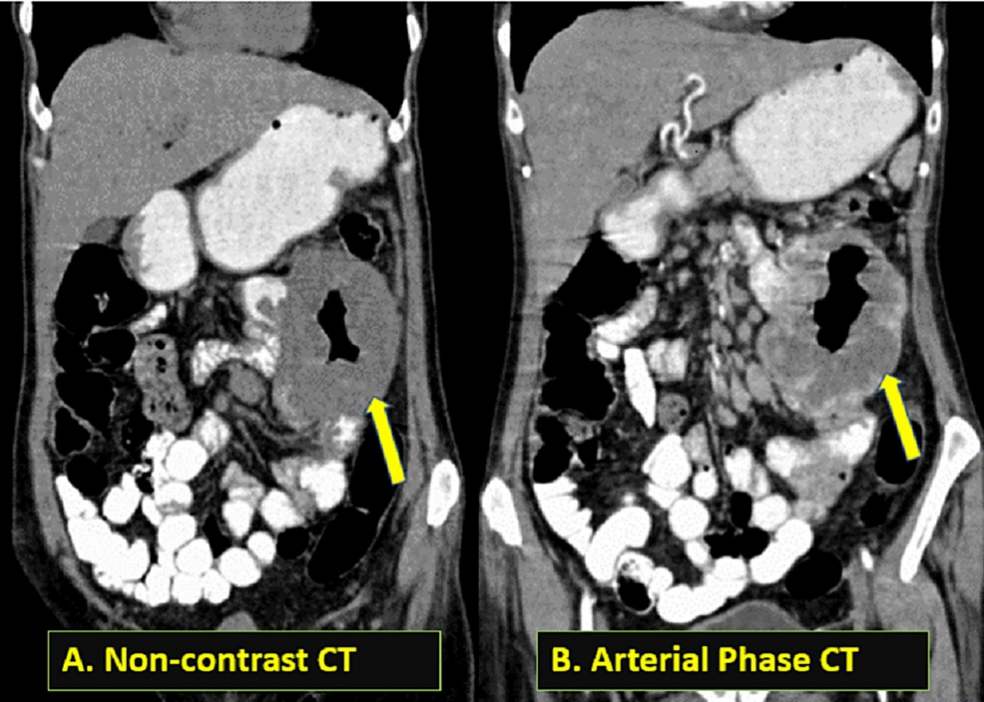
(A) Coronal section, non-contrast computed tomography image (soft tissue window) showing a heterogeneous mass lesion (shown by the arrow) with central necrosis in the left hypochondrium abutting the jejunum with no signs of intestinal obstruction. (B) Coronal section, arterial phase-contrast enhanced computed tomography image (soft tissue window) of a mass lesion with peripheral enhancement (shown by the arrow) and multiple enlarged enhancing pre-aortic, para-aortic, and mesenteric lymph nodes.

**Figure 3:**
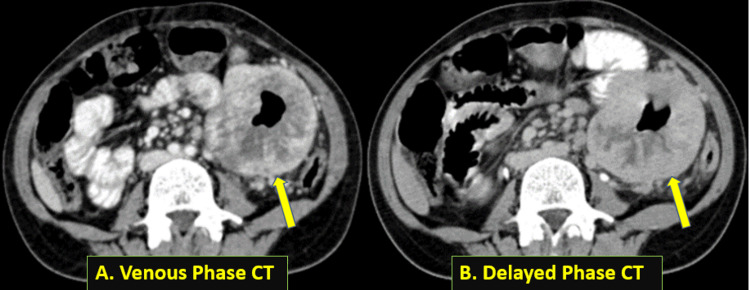
(A) An axial section of a venous phase-contrast enhanced computed tomography image (soft tissue window) demonstrating centripetal filling of the mass lesion with contrast (depicted by an arrow). (B) An axial section of a delayed phase-contrast enhanced computed tomography image (soft tissue window) demonstrating contrast filling of almost the entire lesion (shown by the arrow), with a few non-enhancing areas and central air collection.

On computed tomography (CT) scan, there was a heterogeneous exophytic mass lesion (Figure 2A) abutting the jejunal loop in the left hypochondrium with air density foci within, showing irregular peripheral enhancement in the arterial phase (Figure 2B) and centripetal filling in the venous (Figure 3A) and delayed phases (Figure 3B). The lesion measures approximately 8.5 x 7.8 x 6.5 cm in size. There was no luminal narrowing of the involved jejunum. There were numerous enlarged pre- and para-aortic and mesenteric lymph nodes, the largest of which measured 1.6 x 1.5 x 1.3 cm. There were no identifiable metastases in other abdominal organs or bones.

**Figure 4:**
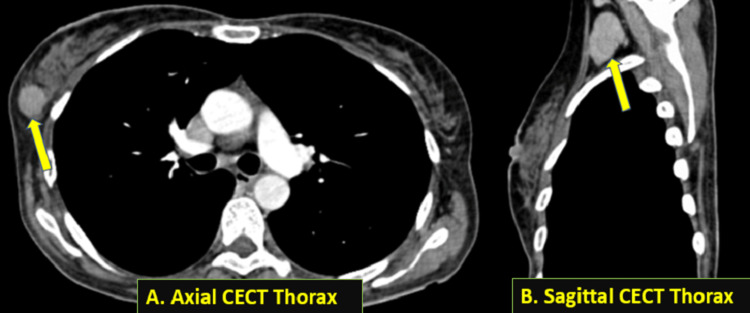
(A) Axial section, arterial phase-contrast enhanced computed tomography (CECT) image (soft tissue window) showing an enhancing mass lesion (shown by the arrow) in the upper outer quadrant of the right breast. (B) Sagittal section, venous phase-contrast enhanced computed tomography (CECT) image (soft tissue window) showing a heterogeneously enhanced enlarged lymph node (shown by the arrow) in the right axilla.

The contrast-enhanced computed tomography (CECT) thorax also showed an enhancing mass lesion in the lower outer quadrant of the right breast (Figure 4A) with heterogeneously enhancing enlarged ipsilateral axillary lymph nodes (Figure 4B).

The patient underwent surgery. The excised lesion, along with the adjacent segment of the jejunal loop, were sent for histopathological examination. Because of the suspicious features of the lymph nodes on USG and clinical features such as hard consistency on palpation, an excisional biopsy of the right breast mass and axillary lymph nodes was also performed. The samples from the breast lump and enlarged axillary lymph nodes sent for frozen section were initially diagnosed as metastases from malignant epithelioid GIST. However, on histopathology, the breast lump specimen showed predominantly lymphoid tissue with evidence of metastatic deposits of epithelial malignancy and an undisturbed periphery of the ductal and lobular structure of the surrounding breast parenchyma. The axillary lymph nodes also showed metastases of epithelial malignancy.

**Figure 5:**
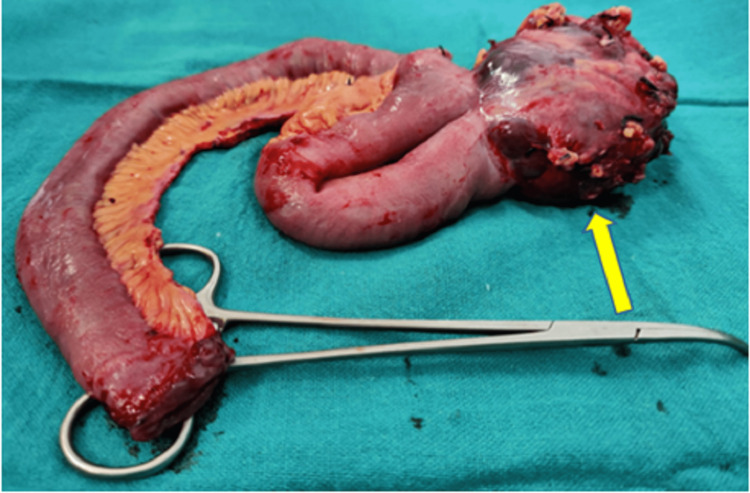
A gross specimen of a surgically resected jejunal loop with a mass lesion arising from its wall (shown by the arrow).

The mass from the resected jejunum (Figure 5) had features consistent with malignant GIST (spindle cell variant), and the proximal and distal margins of the jejunum were negative for infiltration by malignant cells. All the collected specimens from abdominal lymphadenopathy showed reactive hyperplasia and were negative for infiltration by malignant epithelial cells.

On immunohistochemistry, the breast specimen was weakly positive for estrogen receptor (ER), negative for progesterone receptor (PR), and positive for Her2Neu. The GIST specimen was positive for C-KIT, discovered on GIST-1 (DOG-1), and SMA, and negative for S-100 protein. The pathological TNM staging was given as pT3pNxpMx for the GIST, and the patient was started on imatinib therapy. Adjuvant chemotherapy was also advised for the resected breast cancer (locally advanced). As a result, this case demonstrated a unique presentation of GIST with incidental detection of primary breast cancer. 

## Discussion

Gastrointestinal stromal tumors (GIST) are caused by activating mutations of receptor tyrosine kinase (C-KIT proto-oncogene/CD117) present on interstitial cells of Cajal [4]. The most common site of origin for GISTs is the stomach, followed by the small intestine and large intestine in that order, whereas the esophagus is rarely affected. They can also arise from the omentum, mesentery, or retroperitoneum [3,4].

Clinical presentation can vary according to the site of the lesion, size of the lesion, and staging of the disease. Small tumors are mostly asymptomatic, irrespective of the site. Most cases present with non-specific gastrointestinal symptoms such as nausea, loss of appetite, vague pain, and a noticeable lump in larger masses. Bowel obstruction is very rare except in large intraluminal masses. Large tumors can also ulcerate and cause gastrointestinal hemorrhage [3].

Ultrasonographic features include hypoechoic, heterogeneous intraluminal, or extraluminal mass lesions appearing to arise from the wall of the gastrointestinal tract. A typical bull's-eye appearance is seen because of the central area of necrosis seen in larger GISTs. Deep ulceration or central necrosis with an elevated air-fluid level gives rise to the typical Torricelli-Bernoulli sign on CT or MRI [5].

Contrast-enhanced triple-phase computed tomography (CECT) would show peripheral enhancement on the arterial phase with centripetal filling in the venous and delayed phases. Heterogeneity with central necrosis is often seen. Calcifications within the mass are rare. Extra-luminal lesions, which arise from the extra-mucosal layers of the bowel wall with intact mucosa and an unobliterated lumen, rarely cause an intestinal obstruction. MRI characteristics include heterogeneous lesions that can have areas of hemorrhage, necrosis, or cystic change; commonly a hyperintense solid component on a T2 weighted image (T2WI); occasionally hypointense areas on a T2WI that can represent a fibrous component; progressive enhancement on post-gadolinium contrast T1 weighted (T1+C) images; and peripheral enhancement in the case of larger lesions [6]. Large lesions can outgrow their vascular supply and demonstrate central necrosis and less arterial enhancement.

The FDG-PET scan adds to the staging process in GIST. Metastasis in the breast is highly unusual in GIST. Metastasis to the liver, peritoneum, lung, bones, etc., occurs commonly in GIST, whereas metastasis to the lymph nodes and brain occurs less commonly. In order to differentiate the breast metastasis in GIST from other malignant or benign tumors of the breast, like phyllodes tumors, immunohistochemistry for C-KIT protooncogene receptor kinase (CD 117), which was discovered on GIST-1 (DOG-1), is performed [4,7]. The concomitant occurrence of other primary cancers has been reported in the literature, with an incidence of 17% according to a population-based study by Murphy et al., 2015. The concomitant cancers included a wide variety of carcinomas, sarcomas, carcinoids, and lymphomas arising from various organs. However, no increased incidence of breast cancer was found in this study, unlike in previous studies. Overall, concomitant primary cancer of breast origin is very rare [8,9].

The differential diagnosis of GIST includes primary and metastatic lesions of the small intestine. Adenocarcinoma appears radiologically different from GISTs and is seen as an annular lesion. However, gastrointestinal lymphoma, in which there is a predominant thickening of the bowel wall and lymph nodal enlargement, can sometimes have overlapping radiological features, with GISTs showing ulceration and cavitation in the case of large masses. Other differentials include gastrointestinal leiomyomas, which are more common in the esophagus; gastrointestinal leiomyosarcoma, which is a rare entity; gastrointestinal schwannoma, which is more homogeneous and lacks the cystic change seen in GIST; and gastrointestinal carcinoid, which demonstrates mesenteric metastasis and stranding, giving a spoke wheel appearance, and polypoidal or plaque-like growth [10].

Rarely, extraintestinal GIST can arise from the retroperitoneum or mesentery and may not be attached to the bowel wall, in which case the diagnosis becomes challenging. However, the histopathological diagnosis of GIST is confirmed by the presence of sheets of spindle-shaped cells with hyperchromatic nuclei that stain positive for CD117 and were recently discovered in GIST-1 (DOG-1) by immunohistochemistry [4, 10].

Most tumors have already metastasized by the time of diagnosis. However, lymph node enlargement is not very common in GIST but occurs more commonly in younger patients <40 years of age [11]. Surgical resection of the tumor is the primary treatment modality. Because GISTs have a high recurrence rate, imatinib mesylate is used as adjuvant treatment in cases where recurrence is suspected, and the use of imatinib mesylate has dramatically improved the prognosis in such cases. Imatinib mesylate is a tyrosine kinase inhibitor that specifically targets CD117. Newer alternatives to imatinib, such as sunitinib, regorafenib, avapritinib, and ripretinib, have been introduced for treatment-resistant cases. Regular follow-up with radiological investigations and FDG-PET studies is recommended post-surgery [12].

## Conclusions

Gastrointestinal stromal tumors are a distinct entity that can arise anywhere from the esophagus to the anus, as well as in the mesentery, omentum, and retroperitoneum, with characteristic radiological appearances. CD117 and DOG-1 expressions are considered confirmatory for diagnosis when other differentials cannot be ruled out. This case constitutes an interesting and rare presentation of a gastrointestinal stromal tumor with concomitant detection of primary breast cancer. The radiological features of breast masses like homogeneity and regular margins made the diagnosis tricky initially, and immunohistochemistry proved to be highly beneficial in the differential diagnosis. However, early radiological detection of the GIST along with breast and axillary masses aided in the timely management and treatment planning of the patient.
